# Directly wireless communication of human minds via mind-controlled programming metasurface

**DOI:** 10.1038/s41377-022-00831-7

**Published:** 2022-06-16

**Authors:** Xiangang Luo

**Affiliations:** State Key Laboratory of Optical Technologies on Nano-Fabrication and Micro-Engineering, Institute of Optics and Electronics, Chinese Academy of Sciences, P.O. Box 350, Chengdu, 610209 China

**Keywords:** Metamaterials, Photonic devices

## Abstract

An concept of electromagnetic brain-computer-metasurface (EBCM), and remotely mindcontrolled metasurface (RMCM) via brainwaves is reported in *eLight*. Rather than DC voltage from power supply or AC voltages from signal generators, such metasurfaces are controlled by brainwaves collected in real time and can transmit information wirelessly between human brains. Such platforms can lead to a promising approach for the service of disabled people.

Reconfigurable metasurfaces are highly desirable for applications including dynamical camouflaging and intelligent electromagnetic (EM) skin due to their tunable EM features or functionalities^1–3^. Tuning are traditionally achieved by using electric voltage^4^, thermal free carriers^5^, mechanical stretching^6^, or optical illumination^7^.

In recent works published in eLight^8,9^, Ma et al.^8^ and Zhu et al.^9^ casted a roadmap fusing of the reprogrammable EM metasurfaces with Brain-computer interface (BCI), which paved the way towards non-contact and real-time tuning of metasurfaces. With their findings, metasurfaces can be dynamically tuned by brain messages, and wirelessly transmit brain messages between operators.

As reported by^8^ and shown in Fig. 1, the text image in front of Operator A can generate EEG signals and be decoded in ASCII coding sequences. When implemented on FPGA, it can switch time-varying patterns on the transmitter’s metasurface and be wireless transmitted to the receiver’s metasurface, and then translated back into text information in front of Operator B. In the whole process, neither Operator A nor Operator B needs muscle actions. The authors named this platform as electromagnetic brain-computer-metasurface (EBCM). With EBCM, the authors also demonstrated three typical schemes with distinct functions, including visual beam scanning, multiple EM function switching, and metasurface pattern input, which contains more than 20 coding patterns for different single-beam scanning, multi-beam forming, OAM-beam generation, and RCS control.

**Fig. 1:**
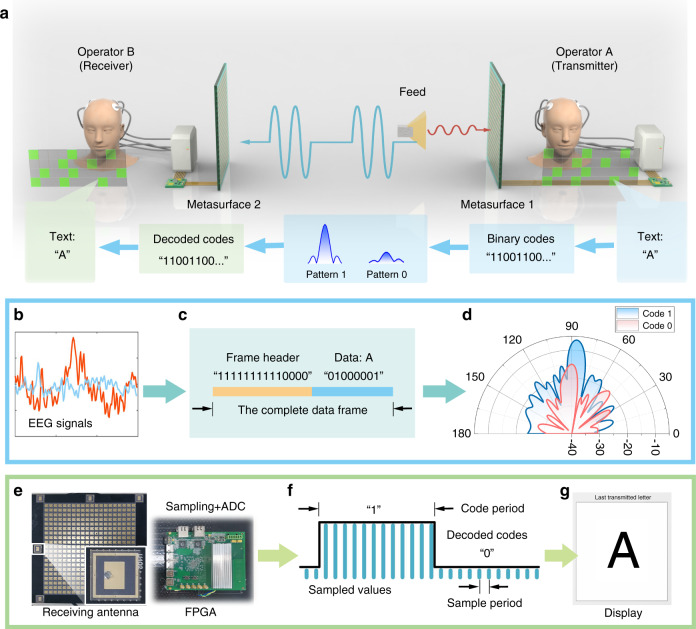
The wireless text-communication using EBCM. **a** The system architecture of the text-communication system as well as the coding and decoding process. The encoding process from EEG signals to the transmitted EM signals, where the EEG signals shown in (**b**) are first detected by BCI and translated into the digital sequence in (**c**) for wireless transmission, then radiated by the metasurface with different pattern amplitudes in (**d**). **e**–**g** The decoding process of wireless communication, where the antenna and FPGA in (**e**) firstly receive and sample the signals from space and convert them into the digital signals. The sampled data are discretized into 0/1 codes for decoding, as depicted in (**f**) and finally translated into the text for display. Reprinted with permission from Ref. ^8^, Copyright (2022)

At the same time, Ref. ^9^ demonstrated a programmable metasurface with coding sequences that are reprogrammed by the human mind to perform different functionalities^9^.

As shown in Fig. 2^9^, the authors proposed a framework for realizing remotely mind-controlled metasurface (RMCM) using a brainwave extraction module. This framework consists of three parts, i.e., sensor, controller, and actuator. The sensor refers to the brainwave module that can record brainwaves and transmit them to the controller via Bluetooth. The controller consists of the microprogrammed control unit (MCU) and an output terminal. Arduino is selected as the controller in this work. The controller receives brainwave signals from the sensor, converts them into attention signals, and then feeds the attention signals into the actuator. According to the level of attention intensity, the attention signals fall within four separate intervals which are characterized by four thresholds. The output pins of the controller are connected to the actuator, and the high/low level of output voltage corresponds to the coding 1/0 sequence. Under the switch on/off state of the PIN diodes, a phase difference of 180° is produced to enable 1-bit coding. The output voltage level sequences from the controller denote different coding sequences on the metasurface, to control the scattering pattern. Both the simulated and test results show that the EM response of metasurface can be directly controlled by the user’s brainwaves. Both EBCM and RMCM show good performances in EM wave modulation, providing a new route to intelligent metasurfaces.

**Fig. 2:**
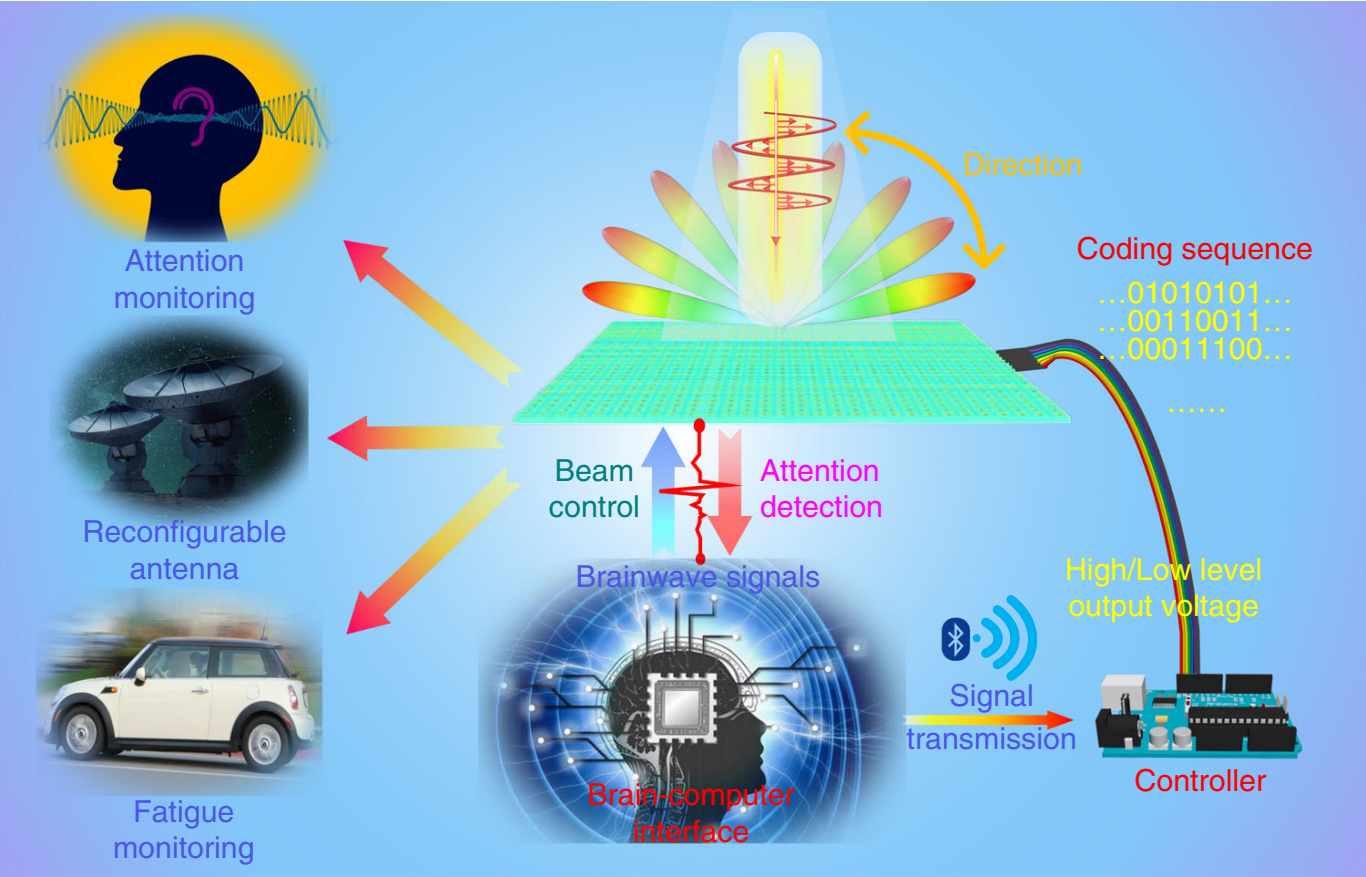
Schematic diagram of remotely mindcontrolled metasurfaces via brainwaves. To achieve remote control, brainwaves signals are transmitted wirelessly from the user to the controller via Bluetooth. A brainwave extraction module is employed to extract the user’s attention intensity signal, which is then used as the control signal of programmable metasurfaces. Reprinted with permission from Ref. ^9^, Copyright (2022)

In comparison with the state-of-the-art tuning methods, the presented new control manner - mind control with brainwaves - is more direct and effective, leading to a promising approach for the service of disabled people. Customized designs for different users will further improve the accuracy of equipment in the future. Combined with intelligent algorithms such as machine learning, the presented two works may further open up a new direction to advanced bio-intelligent metasurface systems.

## References

[1] Huang C (2017). Reconfigurable metasurface cloak for dynamical electromagnetic illusions. ACS Photonics.

[2] Tsilipakos O (2020). Toward intelligent metasurfaces: the progress from globally tunable metasurfaces to software-defined metasurfaces with an embedded network of controllers. Adv. Opt. Mater..

[3] Luo X (2020). Metasurface waves in digital optics. J. Phys. Photonics.

[4] Cui T, Bai B, Sun H (2019). Tunable metasurfaces based on active materials. Adv. Funct. Mater..

[5] Rahmani M (2017). Reversible thermal tuning of all-dielectric metasurfaces. Adv. Funct. Mater..

[6] Ee HS, Agarwal R (2016). Tunable metasurface and flat optical zoom lens on a stretchable substrate. Nano Lett..

[7] Shcherbakov MR (2017). Ultrafast all-optical tuning of direct-gap semiconductor metasurfaces. Nat. Commun..

[8] Ma, Q. et al. Directly wireless communication of human minds via non-invasive brain-computer-metasurface platform. *eLight* **2**, 11 (2022). 10.1186/s43593-022-00019-x.

[9] Zhu, R. et al. Remotely mind-controlled metasurface via brainwaves. *eLight* **2**, 10 (2022). 10.1186/s43593-022-00016-0.

